# Relative white blood cell counts, heterophil-to-lymphocyte ratio, and discovery of blood parasites in wild dugong (*Dugong dugon*) at Lingayan Island, Toli-toli, Indonesia

**DOI:** 10.14202/vetworld.2020.1849-1853

**Published:** 2020-09-10

**Authors:** Aryani Sismin Satyaningtijas, Agik Suprayogi, Huda Shalahudin Darusman, Ardiansyah Nurdin, Desrayni Hanadhita

**Affiliations:** 1 Department of Anatomy Physiology and Pharmacology, Faculty of Veterinary Medicine, IPB University, Bogor, Indonesia; 2 Study Program of Veterinary Medicine, Faculty of Medical, Hasanuddin University, Makassar, Indonesia

**Keywords:** *Anaplasma*, *Babesia*, blood smear, leukocytes, sirenian

## Abstract

**Aim:**

This study was conducted to investigate the relative white blood cell (WBC) counts and the heterophil-to-lymphocyte (H/L) ratio and to analyze the presence of blood parasites in wild dugongs at Lingayan Island. It is expected that the results of this study could provide additional knowledge about the physiological conditions of wild dugongs in their natural habitats, which can serve as basic data in dugong conservation efforts, especially in Indonesia.

**Materials and Methods:**

A wild dugong was captured around Lingayan Island. Blood samples were collected from the saphenous vein, and blood smears were prepared immediately. The blood smears were examined for leukocyte identification, calculation of relative WBC counts, and presence of blood parasites. The H/L ratio was calculated based on the obtained relative WBC counts.

**Results:**

The relative WBC counts included heterophils 19.4%, lymphocytes 76.4%, and monocytes 3.6%, and the H/L ratio was 0.25. Intraerythrocytic parasites were identified and suspected to be *Anaplasma* and *Babesia*.

**Conclusion:**

This study reports leukocyte values from free-ranging dugongs captured in Lingayan Island, Indonesia. Based on the H/L ratio, the dugong examined, in this study, did not experience chronic stress. However, the discovery of blood parasites could be one of the threatening factors for the dugong population.

## Introduction

Dugongs (*Dugong dugon*) are members of sea cows (Sirenians), which are pure herbivores and their survival depends on seagrass populations. *D. dugon*, which belongs to the subfamily Dugonginae, is the only species of Dugongidae family that still exists [1]. Worldwide, all sirenians, including dugongs, are categorized as vulnerable on the International Union for Conservation of Nature (IUCN) red list, and their population has decreased over time [2]. Various factors, including habitat degradation, hunting, fishing pressure, and disease, contribute to population decline. Being herbivorous marine mammals, dugongs have an important role in maintaining seagrass ecosystems on the coast. The dugong digs and turns the sand for its food, thus maintaining the nutrients required for seagrass. Dugongs also act as a source of nitrogen needed by seagrasses and benthos in coastal areas. The presence of dugongs on the coast can be a biological indicator of a balanced ecosystem [3]. Indonesia is a country consisting of thousands of Islands and also one of the native dugong-origin countries. The Islands of Indonesia provide marine conservation areas comprising a total area of 164,511 km^2^. Moreover, Indonesia is surrounded by the Indian Ocean and the Pacific Ocean so that it becomes an important focal area for dugongs from Southeast Asia, Pacific Islands, and Australia for foraging [4]. One area of Indonesia with a vast expanse of seagrass meadow is on Lingayan Island, Ogotua Village, Toli-toli Regency, and Central Sulawesi Province. Lingayan Island is one of the dugong conservation centers established by the government in coordination with local residents [5,6]. Lingayan Island waters have a temperature of around 30°C, which is suitable for the foraging and breeding of dugongs [7].

Wildlife conservation efforts require an understanding of the physiological response of animals to their habitat. The parameters of conservation can be addressed through physiological approaches, especially those related to stress [8]. Physical and blood examinations are some of the parameters commonly performed for collecting physiological data. A previous study reported about the basic physiological parameters (i.e., heart rate, respiratory rate, and body temperature) of wild dugongs captured on Lingayan Island in June 2018 [7]. A simple blood examination on wildlife that is popular among ecologists and can describe stress conditions in animals is the relative white blood cell (WBC) count estimated using blood smears. Calculation of the heterophil-to-lymphocyte (H/L) ratio is also one of the reliable methods for evaluating the chronic stress response experienced by vertebrates [8].

This study was conducted to examine the relative WBC counts and the H/L ratio and to analyze the presence of blood parasites in wild dugongs at Lingayan Island. We expect that our study findings could provide additional knowledge regarding the physiological conditions of wild dugongs in their natural habitats, which can serve as basic data in dugong conservation efforts, especially in Indonesia.

## Materials and Methods

### Ethical approval

Dugong handling was carried out safely under the supervision of a team of veterinarians following the capture method suggested by Lanyon *et al*. [9]. The procedure for animal restrain and blood collection was approved by The Natural Conservation Agency and The Fisheries Agency in Central Celebes Province as well as The Indonesian Institute of Science (LIPI).

### Study period, animal and environmental condition

**Figure-1:**
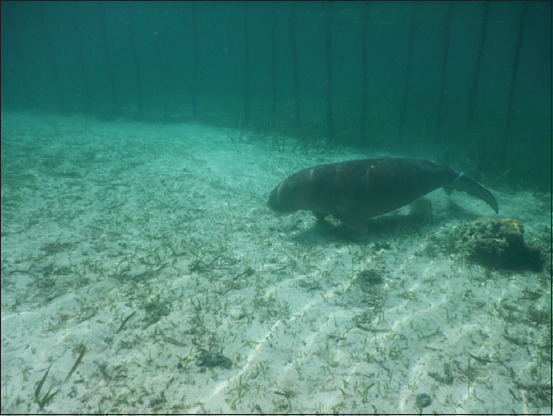
Dugong adaptation in a circular cage made of wood and net.

This study was conducted in August 2019 on the coast of Lingayan Island, Ogotua Village, Toli-toli Regency, Central Sulawesi, Province, Indonesia. A dugong was captured and adapted in a cage for 14 days. The location of the cage was set below the sea level with a distance of approximately 1 km from the beach. The cage was made of wood and net with an area of approximately 1900 m^2^ (Figure-1). The dugong had access to seagrass beds inside the cage.

### Blood sampling and preparation

The blood sampling was conducted on a boat near the coast. The dugong was positioned in dorsal recumbency on a foam mattress that was frequently moistened with water. The blood was drawn from the saphenous vein in the tail. The blood smears were prepared immediately after blood withdrawal. The blood smears were labeled, air-dried, fixed with methanol, and stained with Giemsa.

### Blood smear examination

The blood smears were examined to identify leukocytes, calculate the relative WBC value, and determine the presence of blood parasites. The relative WBC value was calculated by counting 100 leukocyte cells in the blood smear, followed by calculation of each percentage of leukocytes. The presence of blood parasites was examined under a microscope at 100× of at least 50 fields per slide.

### Statistical analysis

Data were analyzed descriptively. The obtained data were compared with the hematology data of dugongs reported in the literature [10].

## Results

**Figure-2:**
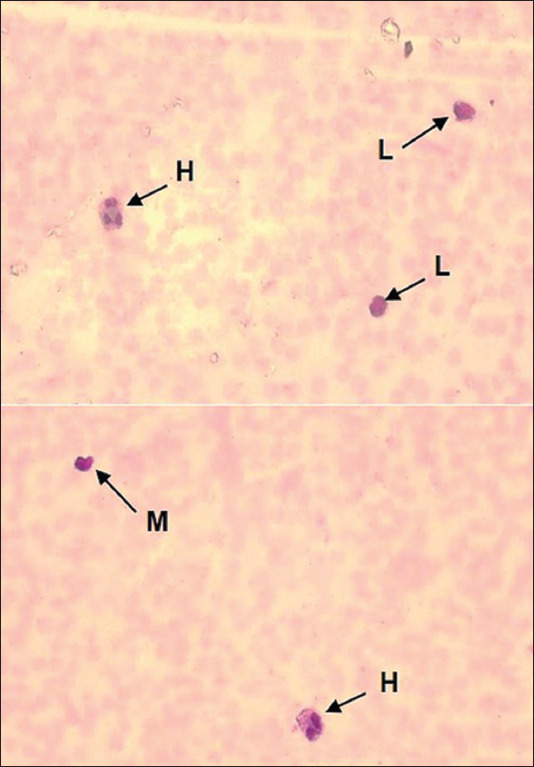
Dugong blood smear preparations. Leukocytes identified in blood smear include heterophils (H), lymphocytes (L), and monocytes (M).

**Figure-3:**
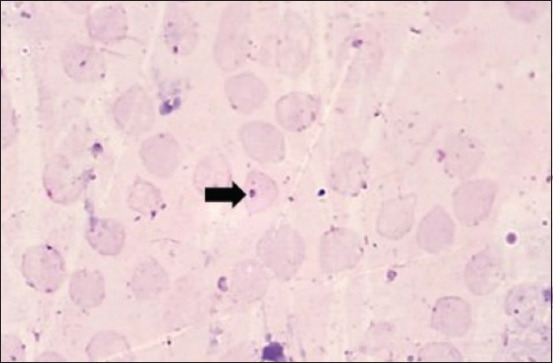
A dark purple intraerythrocytic blood parasite in the form of coccoid, which was suspected to be *Anaplasma* (arrow), was identified on a blood smear.

**Figure-4:**
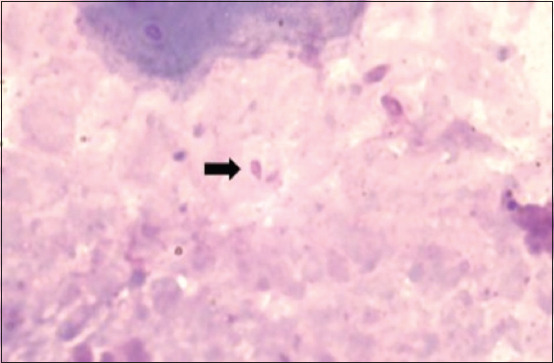
A dark purple pear-shaped intraerythrocytic blood parasite, which was suspected to be *Babesia* (arrow), was identified on a blood smear.

Based on the manual blood smear examination, the relative WBC value was calculated, with heterophils at 19.4%, lymphocytes at 76.4%, and monocytes at 3.6% (Figure-2). Eosinophils and basophils were not detected. The H/L ratio was 0.25. The blood smear examination also demonstrated the presence of dark purple microorganisms inside the red blood cell (RBC) cytoplasm that appeared as coccoid- (Figure-3) and pear-shaped (Figure-4).

## Discussion

There is a scarcity of information regarding the physiological parameters of wild dugong in its habitat. Direct examinations of live dugongs require team and equipment preparations to minimize stress exposure to the dugongs. The blood smear examinations done in the present study showed that the leukocytes and erythrocytes of this dugong had the same morphology as that of mammals in general. However, a previous study described that based on the cytochemical characteristics, the heterophils of dugongs and other sirenians are more classified as heterophils than as neutrophils [10]. Dugongs also have the largest sized erythrocytes compared to those of other marine mammals [10]. The present study also showed that the majority of leukocytes found in the peripheral circulation are lymphocytes, which are in accordance with the previous study [10]. However, the majority of mammals have a higher neutrophil population in the peripheral circulation than other types of leukocytes [8].

The H/L ratio was calculated to determine whether the dugong experienced a physiological impact due to the stress exposure that may occur in the Lingayan Island area. The H/L ratio for the captured dugong was 0.25, whereas the previous study reported an H/L ratio of 0.8 for wild dugongs (n=92) in Australia [10]. The H/L ratio is an accurate parameter that is often used to evaluate the incidence of chronic stress [8,11]. This measurement is considered to be suitable for assessing the behavioral stress responses in free-ranging animals, which is because the ratio reflects the changes in cortisol levels [8,12-14]. Measurement of cortisol cannot be frequently performed due to an extremely rapid increase in its titer values during stress exposure that requires rapid sampling and efficient animal handling. Conversely, changes in leukocyte levels due to increased cortisol levels can be detected after several hours or even days, depending on the vertebrate taxon [8]. Stress triggers increased production of the cortisol hormone in the bloodstream through the hypothalamic-pituitary-adrenal (HPA) axis mechanism. Cortisol decreases the number of circulating lymphocytes by increasing the adhesion of lymphocytes to the blood vessel endothelium and facilitating the transmigration of lymphocytes to the storage organ. However, cortisol also increases heterophil migration from the bone marrow to the bloodstream and prevents heterophil transfer from blood to other compartments. Therefore, the stress response determined from peripheral blood vessels includes neutrophilia and lymphopenia [15]. This results in an increased H/L ratio compared to that in normal conditions. The H/L ratio of the dugong in Lingayan Island did not exceed the previously reported value [10], indicating that this dugong was not under stress. In fact, it has been reported that dugongs are not easily affected by capture stress when capture management is appropriate [16]. In addition to the H/L ratio, fecal glucocorticoid (fGC) measurements can be conducted to evaluate the stress response of dugongs to the stress environment of their habitat without causing stress due to the capture process. Female pregnant dugongs and adult males have been reported to have the highest baseline fGC levels, indicating that these two categories are exposed to the greatest stress factor [17]. As Lingayan Island is a dugong conservation site, there are no threats of hunting in this area. In addition, Lingayan Island has a tropical climate that has a constant temperature range throughout the year. Changes in temperature have been reported to influence the fluctuations in glucocorticoid levels in dugongs and reduce body scores during winter [17].

In the present study, the blood smear examination also demonstrated the presence of intraerythrocytic dark purple microorganisms appearing as coccoid- and pear-shaped. The coccoid-shaped intraerythrocytic parasites were generally identified as *Anaplasma*, bacteria belonging to the order Rickettsiales, whereas the pear-shaped microorganisms were generally identified as *Babesia*, protozoa belonging to the order Piroplasmida [18-21]. The genus and species of these blood parasites remain unidentified as of now. Further investigations for identifying the genus of these blood parasites in dugongs, including molecular and ultrastructural studies, are required to substantiate our findings. Micro-endoparasites that have been reported to infect dugongs include *Toxoplasma*, *Neospora*, and *Cryptosporidium* [22-24]. The discovery of intraerythrocytic blood parasites in the present study is an interesting finding as, to the best of our knowledge, it has not been reported in dugongs till date.

*Anaplasma* and *Babesia* are the intraerythrocytic parasites that commonly infect mammals throughout the world [18-20]. The vectors of these two parasites are generally the blood-sucking arthropods [25,26]. However, there are no reports of blood-sucking arthropod infestations in dugongs. Ectoparasites reported in dugongs are generally considered as commensal [3]. There are also several reports of severe trematode infections found in dugong carcasses [24]. Trematodes are also a vector of *Neorickettsia*, which are the members of the Anaplasmataceae family of bacteria [27,28]. Further research is needed to assess whether there is a link between trematode infection and intraerythrocytic parasite infection in dugongs.

Anaplasmosis and babesiosis events are often subclinical in wild animals. However, severe infections can occur when animals experience stress conditions [29,30]. Anaplasmosis and babesiosis can cause hemolytic anemia, and in cases of severe infections can result in death. *Anaplasma* and *Babesia* can be transmitted vertically, through trans uterus, and from parent to fetus [31]. Severe infections of anaplasmosis and babesiosis can be fatal for newborns. Dugongs give birth to only a single calf at intervals of 3-7 years [3]; therefore, a blood parasite infection can threaten the population. Further studies must be conducted to understand the nature of blood parasite infections in dugongs.

Lingayan Island, which is a part of the Central Sulawesi province, is one of the outer Islands of Indonesia. This Island is surrounded by vast seagrass beds with various types of seagrasses that are highly nutritious for dugongs [5]. The availability of food and warm water temperatures make the Lingayan coast a comfortable habitat for dugongs [5-7]. Therefore, in the efforts aimed at dugong conservation, it is necessary to protect their natural habitat ecology and also prevent the occurrence of infectious diseases.

## Conclusion

The morphology of leukocytes and erythrocytes in dugongs was similar to that in mammals in general. The majority of leukocytes found in the peripheral circulation are lymphocytes. Based on the H/L ratio, the dugong in Lingayan Island did not experience chronic stress due to environmental factors. Intraerythrocytic dark purple microorganisms were identified in the blood smear, which were suspected to be blood parasites belonging to the genera *Anaplasma* and *Babesia*.

## Authors’ Contributions

ASS conceived the idea. ASS, AS, HSD, and AN were involved in research design and research data collection. ASS, AS, HSD, AN, and DH were involved in data analysis and manuscript writing. All authors read and approved the final manuscript.
